# GEFT inhibits the GSDM-mediated proptosis signalling pathway, promoting the progression and drug resistance of rhabdomyosarcoma

**DOI:** 10.1038/s41419-024-07243-y

**Published:** 2024-11-30

**Authors:** Fan Yang, Tian Xia, Zhijuan Zhao, Jinyang Lin, Ling Zhong, Tian Tang, Degui Liao, Miaoling Lai, Jiamin Ceng, Lian Meng, Feng Li, Chunxia Liu

**Affiliations:** 1 Department of Pathology, The Second Affiliated Hospital of Guangzhou Medical University, Guangzhou, 510260 China; 2 Department of Pathology, Shihezi University School of Medicine and The Key Laboratories for Xinjiang Endemic and Ethnic Diseases, Chinese Ministry of Education, Shihezi, 832002 Xinjiang China; 3 Medical Research Center & Department of Pathology, Beijing Institute of Respiratory Medicine and Beijing Chao-Yang Hospital, Capital Medical University, Beijing, 100020 China

**Keywords:** Sarcoma, Sarcoma

## Abstract

The metastasis or recurrence of rhabdomyosarcoma (RMS) is the primary cause of tumour-related deaths. Patients with high-risk RMS have poor prognosis with a 5-year overall survival rate of 20–30%. The lack of specific drug-targeted therapy and chemotherapy resistance are the main reasons for treatment failure. Drugs or molecular target inhibitors can induce the pyroptosis of tumour cells or increase their sensitivity to chemotherapy, making pyroptosis an effective strategy for antitumour therapies. Pyroptosis is mediated by gasdermin (GSDM) family members. Here, we found that the expression of NLRP3, caspase-1, caspase-3, GSDMD and GSDME in RMS was remarkably lower than that in skeletal muscle tissues. Nigericin and dactinomycin in RMS cells achieved their regulatory effect on pyroptosis through the NLRP3/caspase-1/GSDMD pathway and caspase-3/GSDME pathway, respectively. Necrosulfonamide reversed the pyroptosis-related changes induced by nigericin, and siGSDME converted the dactinomycin-induced pyroptosis into apoptosis. Additionally, GEFT inhibited the GSDMD and GSDME pyroptosis pathways, thereby promoting the progression and drug resistance of RMS. Mouse xenograft and tumour analysis confirmed that nigericin and dactinomycin can effectively improve the therapeutic effect of RMS by activating the pyroptosis pathway. To the best of our knowledge, this study was the first to focus on pyroptosis in RMS. Overall, our investigation demonstrated that nigericin and dactinomycin play therapeutic roles in tumours by promoting RMS cell pyroptosis. Interference with GEFT and drug combination can exert a great inhibitory effect on tumours.

## Introduction

Rhabdomyosarcoma (RMS) is the most common soft-tissue sarcoma in children. Despite multimodal therapies, patients with high-risk RMS have poor prognosis with a 5-year overall survival rate of 20–30% [1]. Alveolar RMS (ARMS), embryonic RMS (ERMS), pleomorphic RMS (PRMS) and spindle cell/sclerosing RMS (SRMS) are the major subtypes of RMS. Novel RMS subtypes characterised by EWSR1/FUS::TFCP2 gene fusions or MEIS1::NCOA2 gene fusion have been recently described. ARMS, MYOD1-mutant SRMS, PRMS, and RMS with TFCP2-associated gene fusion often have a poor prognosis [2, 3]. From a genetic perspective, RMS may be a fusion-positive (FP) or fusion-negative (FN) subtype. FP RMS is associated with PAX3/7-FOXO1 or EWSR1/FUS::TFCP2 fusion gene, and FN RMS involves signalling pathways, including hedgehog, Notch and Wnt signalling pathways [4]. Epigenetic mechanisms regulate gene expression and contribute to RMS development. Genome-wide DNA methylation studies report evidence of the relationship of DNA methylation with mutational changes and transcriptional organisation in FP and FN RMS [5, 6]. Therefore, the pathogenesis of RMS involves many changes in genes, signalling pathways and epigenetics, leading to a poor treatment effect.

Pyroptosis is a new procedural cell death mediated by gasdermin (GSDM) family members, including gasdermin A (GSDMA), gasdermin B (GSDMB), gasdermin C (GSDMC), gasdermin D (GSDMD), gasdermin E (GSDME) (also known as DFNA5) and PJVK (also known as DFNB59) in humans [7, 8]. GSDMD and GSDME are the two most important pyroptosis proteins. GSDMD primarily participates in canonical and noncanonical inflammasome pyroptosis pathways. In the former, pathogen-associated or damage-associated molecular patterns stimulate inflammasomes, followed by inflammasomes activating caspase-1, which is involved in the maturation of the pro IL-18 and pro IL-1β. Inflammatory reactions and GSDMD cleavage subsequently occur [9]. The GSDMD-N fragment releases and forms the pores in the plasma membrane, inducing cell swelling and osmotic lysis. In the latter, lipopolysaccharides derived from bacteria recognise and activate caspase-4/5/11, inducing pyroptosis through GSDMD cleavage [10]. The apoptotic caspase-mediated pyroptosis pathway activates caspase-3 and induces GSDME cleavage, generating GSDME-C and GSDME-N fragments. GSDME-N fragment perforates membranes and thus induces pyroptosis [11]. Owing to its tightly structured signalling cascades and molecular mechanisms, resisting cell death may uncover novel therapeutic targets. Pyroptosis, necroptosis and ferroptosis have been deemed crucial to cancer therapy effectiveness [12, 13]. However, the involvement of pyroptosis in RMS progression remains unclear.

Drugs can induce the pyroptosis of tumour cells and increase their sensitivity to chemotherapy as an effective strategy for antitumour therapies [11, 14]. GSDME cleavage by caspase-3 determines lobaplatin-induced pyroptosis in colon cancer cells [15]. The combination of DNA demethylation and chemotherapy can be an effective strategy for lung cancer treatment via the pyroptosis mechanism [16]. Nigericin is an antibiotic extracted from *Streptomyces hygroscopicus*. It has potential therapeutic properties against a variety of cancers [17, 18], but its effect on RMS has never been reported. Dactinomycin is a chemotherapy agent for patients with RMS and has been studied in a multicentre and phase-3 trial [19, 20], however, its specific mechanism has not been explored. Whether nigericin or dactinomycin plays an important role in RMS by inducing pyroptosis is currently unknown.

In this study, we present evidence suggesting that pyroptosis plays an important role in RMS. Our findings indicate that nigericin and dactinomycin achieve their regulatory effect on pyroptosis in RMS through the NLRP3/caspase-1/GSDMD pathway and caspase-3/GSDME pathway, respectively. Guanine nucleotide exchange factor T (GEFT) can inhibit these pathways by promoting the progression and drug resistance of RMS. Moreover, interference with GEFT and drug combination can exert a great inhibitory effect on tumours. Mouse xenograft and tumour analysis confirmed that nigericin and dactinomycin can effectively inhibit tumour growth by activating the pyroptosis pathway, rendering them potentially effective drugs against RMS.

## Materials and methods

### Human tissue samples

Eight formalin-fixed paraffin-embedded (FFPE) RMS and twenty normal skeletal muscle tissues were selected from the archives of the Department of Pathology of the Second Affiliated Hospital of Guangzhou Medical University. The eight cases of RMS included 4 cases of ERMS, 2 cases of SRMS, 1 case of ARMS and 1 case of PRMS. Written informed consent was obtained from the patients. This study was conducted in accordance with the recognised ethical guidelines approved by the Second Affiliated Hospital of Guangzhou Medical University and the Declaration of Helsinki (2023hs0102).

### Cell cultures and transfection

The following three cell lines derived from human RMS were purchased from the Biological Technology Co., Ltd. (Fu Xiang, Shanghai, China): RD (ERMS, RRID: CVCL_1649), RH30 (ARMS RRID: CVCL_0041) and PLA802 (ARMS, RRID: CVCL_AX34). A normal skeletal muscle cell line (HSKMC) was purchased from the American Type and Culture Collection (ATCC, USA). The cells were cultured in DMEM (Gibco, C11995500BT) medium containing 10% foetal bovine serum and 2 mM l-glutamine. The cell lines were authenticated by short tandem repeat profiling. Mycoplasma testing was performed monthly using a PCR Mycoplasma Detection Kit. RMS cells were grown on six-well plates for transfection with lipofectamine 2000 (Life Technologies, USA) following the manufacturer’s instructions.

### Antibodies, inhibition and drugs

The main primary antibodies for Western blotting were GEFT (Abcam Cat# ab127690, RRID:AB_11127677), Rac1 (Abcam Cat# ab33186, RRID:AB_777598) and Cdc42 (Abcam Cat# ab187643, RRID:AB_2818943), GSDMD (Abcam Cat# ab210070, RRID:AB_2893325), GSDME (Abcam Cat# ab215191, RRID:AB_2737000), caspase-1 (Abcam Cat# ab179515, RRID:AB_2884954), cleaved caspase-1 (Cell Signalling Technology Cat# 4199 (also 4199S, 4199T, 4199P), RRID:AB_1903916), caspase-3 (Cell Signalling Technology Cat# 9662, RRID:AB_331439), cleaved caspase-3 (Cell Signalling Technology Cat# 9664 (also 9664P), RRID:AB_2070042), cleaved PARP (Abcam, ab32064), NLRP3 (Abcam Cat# ab4207, RRID:AB_955792); (Cell Signalling Technology Cat# 15101, RRID:AB_2722591) and β-actin (ZSGB-Bio, China). For immunofluorescence (IF), the primers were caspase-1 (Thermo Fisher Scientific Cat# PA5-17570, RRID:AB_10987516), GSDMD (Thermo Fisher Scientific Cat# PA5-104324, RRID:AB_2853634), FITC-labelled goat anti rabbit (ZSGB-Bio Cat# ZF-0311, RRID:AB_2571576) and TRITC-labelled goat anti mouse (ZSGB-Bio Cat# ZF-0313, RRID:AB_2571577). For immunohistochemistry (IHC), the primers were GSDMD (Proteintech Cat# 20770-1-AP, RRID:AB_10696319), GSDME (Proteintech Cat# 13075-1-AP, RRID:AB_2093053), caspase-1 (Santa Cruz Biotechnology Cat# sc-56036, RRID:AB_781816), caspase-3 (Thermo Fisher Scientific Cat# MA5-11516, RRID:AB_10983525) and NLRP3 (Proteintech Cat# 19771-1-AP, RRID:AB_10646484). The secondary antibodies were peroxidation-conjugated goat anti-mouse/rabbit IgG (ZSGB-Bio Cat# ZB-2301, RRID:AB_2747412), (ZSGB-Bio Cat# ZB-2305, RRID:AB_2747415) and poly-HRP goat anti-rabbit/mouse IgG (IbP, China). Dactinomycin (Selleck, S8964), nigericin (MEC, HY-100381), NSA (Abcam, ab143839), NSC23766 (Selleck, S8031) and ZCL278 (Selleck, S7293) were acquired commercially.

### IHC staining and slide scores

IHC staining of FFPE tissues was performed in accordance with the antibody instructions. Caspase-1, GSDMD, GSDME and NLRP3 were positively detected in the cytoplasm, and caspase-3 was positively detected localised in the cytoplasm and/or nucleus. IHC scoring was based on the proportion of positive cells and the degree of staining. The degree of staining score was negative (0), light brown yellow (1), tan (2) and brown (3); and the staining area score was 0 (0–5%), 1 (6–25%), 2 (26–50%), 3 (51–75%), and 4 (76–100%). Multiplying the two scores yielded the final score. The mean score from ten different high fields was determined as the final IHC score. Scores ≥ 6 were classified as high expression, and scores below 6 were classified as low expression [21].

### Light and electron microscope observation

The cells were seeded onto six-well plates for culture and treated with various specified treatments 12–24 h later. Cell morphology was photographed by light microscopy (Olympus IX71), and the images were saved after the shooting. The cells were fixed in glutaraldehyde fixative for 2 h, dehydrated using an ethanol solution gradient, dried using supercritical fluid and sprayed with gold before observing under a scanning electron microscope.

### SiRNA interference experiments

siRNAs were purchased from GenePharma Company and transfected into RD and RH30 cells. The cells were inoculated on a six-well plate at a density of 3 × 10^5^ cells per well, and adhesion was allowed overnight. After the medium was changed to a serum-free medium, siRNA and lipofectamine 2000 were added to the medium. The medium was replaced with conventional growth medium after 6 h, and the cells were harvested 48 h after transfection.

### RNA isolation and quantitative real-time PCR (qRT-PCR) analyses

The total RNA of the tissues and cells was isolated using TRIzol Reagent. Complementary DNA (cDNA) was synthesised using an RT Reagent Kit (Fermentas K1622). Gene primers were purchased from GenePharma Company, combined with SYBR Green PCR Kit (QIAGEN) and tested in ABI 7500 real-time polymerase chain reaction thermal cyclometer (Applied Biosystems). GAPDH served as standardised control. The primer sequences used in the qRT-PCR analysis can be found in Table S1.

### Western blot analysis

The treated cells were lysed in RIPA lysis buffer (Solarbio). An equal amount of total protein was loaded onto a 10% SDS-polyacrylamide gel and transferred into a polyvinylidene fluoride membrane after electrophoresis. The grey value of the bands was analysed using Image J software.

### Determination of lactate dehydrogenase (LDH)

The LDH released from cell-culture supernatants were detected using a ELISA kit from Westang Biotech, Shanghai, China. The absorbance value for each sample was measured at 490 nm following the manufacturer’s recommended protocol.

### Cell proliferation assays

Cell proliferation was monitored by Cell Counting Kit-8 (CCK8) assay (Dojindo, Shanghai, China) and EdU (KGA337, KeyGen BioTECH, China) assay. The cells were inoculated in a 96-well culture plate and then subjected to the corresponding treatments. Following the manufacturer’s protocol, 10 µL of CCK-8 reagent was added to each well for 2 h. An absorbance was measured at 450 nm with a microplate reader to calculate cell proliferation. For the EdU assay, the cells were cultured on 12-well plates at a cell density of 1 × 10^5^ cells/well. Cell proliferation was assessed every 48 h in accordance with the manufacturer’s protocol.

### Colony-formation assay

The cells were seeded onto six-well plates (1000 cells per well) and incubated overnight at 37 °C and 5% CO_2_. They were then treated with dactinomycin or nigericin for 6 h, and the medium was replaced with a fresh one. After 14 days of culture, the cells were fixed with 4% paraformaldehyde for 20 min and stained with crystal violet for 20 min.

### Migration and invasion assays

Migration/invasion assays were performed with Transwell® chambers (Corning Inc., USA) following the manufacturer’s protocol. For migration, a culture medium containing 10% serum was added to the lower chamber and a cell suspension was added to the upper chamber. Culturing was continued in the incubator for 24 h. For invasion, the Matrigel^TM^ matrix was used to coat the basement membrane (BD Biosciences). The number of cells that moved from the upper chamber through the membrane plus Matrigel to the lower chamber over 48 h was counted.

### Flow cytometry

The cells were seeded onto a six-well plate and subjected to various treatments. They were collected, washed twice with PBS and stained using an Annexin V-FITC/PI Apoptosis Detection Kit (Abmaking) in accordance with the manufacturer’s instructions. Finally, the stained cells were analysed using a flow cytometer (PARTEC, Germany) with FlowJo 7.6 software.

### IF assay

Slides were immersed with PBS in a culture plate, fixed with 4% paraformaldehyde for 15 min and permeated with 0.5% Triton X-100 for 20 min. Normal goat serum was added to the slides and sealed for 30 min. The primary antibody was added to the slides, which were placed in a wet box for overnight incubation at 4 °C. On the 2nd day, the diluted fluorescent secondary antibody was dropwise added to the slides, which were then placed in a wet box for incubation for 1 h. The PBST-soaked sections were added with DAPI drops and incubated for 5 min to avoid light. Finally, the tablets were sealed with a sealing solution containing an anti-fluorescence quenching agent and observed and photographed under a fluorescence microscope.

### Acridine orange staining (AO)

The cells were seeded onto six-well plates and collected into Eppendorf tubes according to grouping before the experiment. A staining system was constructed so that the volume of cell suspension and AO was 100 ml. After being protected from light for 15 min, the machine was inspected and pictures were quickly taken.

### MTT assay

The cells were seeded onto 96-well plates. MTT solution (20 Μl per well) was added after 24 h of incubation. After 4 h, 150 ml of DMSO was added to each well, and the OD value was detected on the machine after 3 h of reaction in the incubator. A curve was drawn.

### Xenograft studies

Athymic nude mice (BALB/ C, female, 4–6 weeks old, weighing 15–22 g) were purchased from Guangdong Medical Experimental Animal Centre. RD and RH30 cells stably transfected with lentivirus GEFT with green fluorescent protein (GFP) were injected into the left back of the nude mice. The mice were randomly divided into three groups of 8: stably transfected with lentivirus GEFT group (control), dactinomycin group and nigericin group. The mice were monitored every other day, and tumours were measured with callipers. When the tumour length exceeded 5 mm, dactinomycin and nigericin were injected into the tumour. No animals died during the experiment. On day 22, the animals were sacrificed, and the IVIS imaging system (Caliper Life Sciences) was photographed. The tumour tissues were treated in liquid nitrogen or FFPE. The FFPE tumour tissues underwent haematoxylin and eosin (HE) stain and IHC staining. All animal studies were conducted in accordance with the guidelines approved by the Animal Protection and Use Committee of the Second Affiliated Hospital of Guangzhou Medical University (B2022011).

### Statistical analysis

All experiments were carried out at least three times independently. Data are expressed as the mean ± standard deviation (SD) of three independent experiments. Statistical analysis was performed using GraphPad Prism (GraphPad Software Inc., RRID: SCR_002798). One-way ANOVA, Tukey post hoc test and unpaired Student’s *t* test were used to analyse the statistical significance between multiple groups and between two groups, respectively. P < 0.05 was considered statistically significant. *P < 0.05, **P < 0.01 and ***P < 0.001 were noted.

## Results

### Significantly lower levels of pyroptosis in RMS tissue samples and cells

**Fig. 1:**
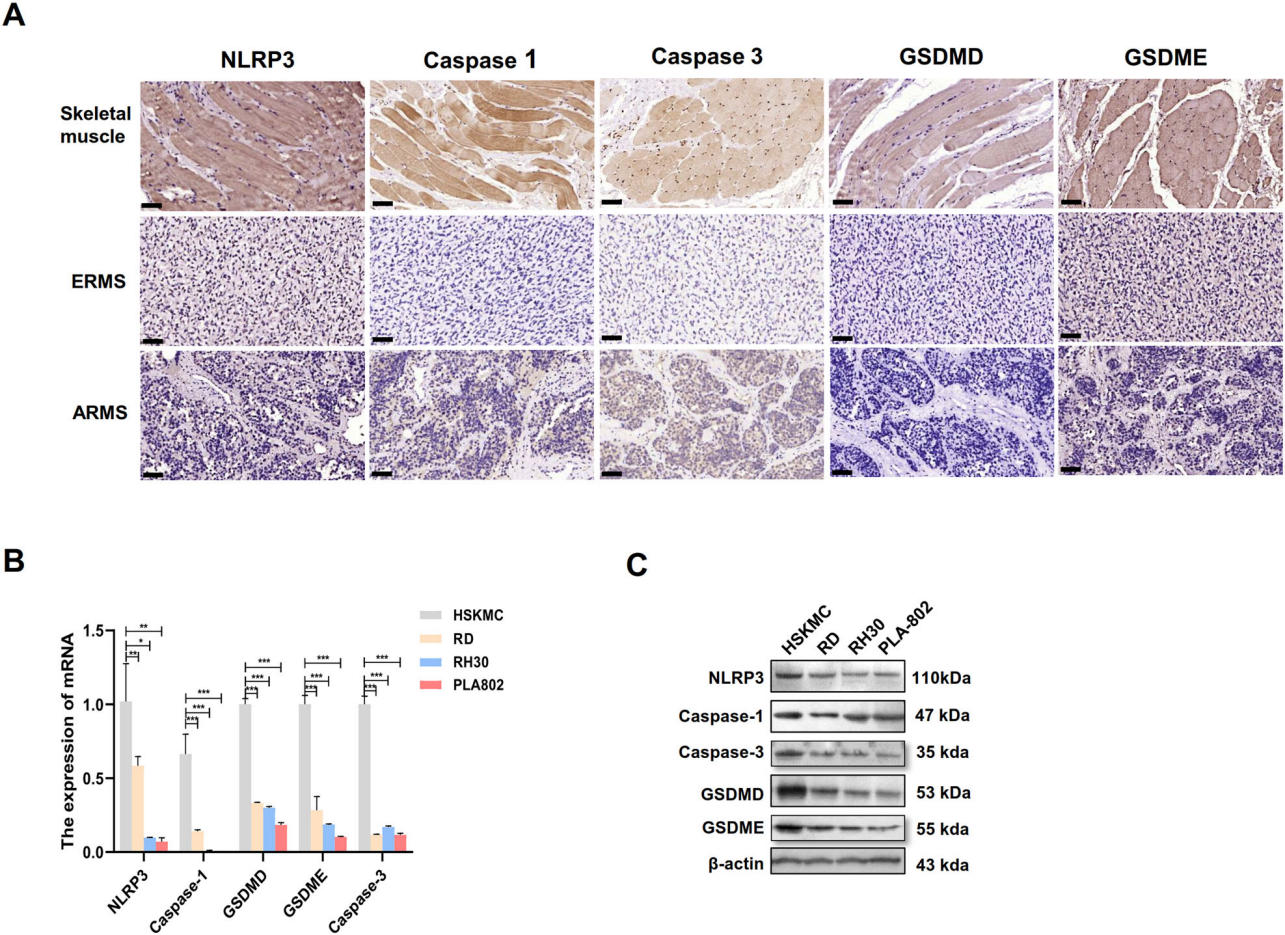
Pyroptosis level was significantly decreased in rhabdomyosarcoma. **A** IHC was used to detect the expression of NLRP3, caspase-1, caspase-3, GSDMD and GSDME in RMS tissues and normal skeletal muscle tissues. Scale bar, 100 µm. **B** qRT-PCR assay was used to detect the mRNA expression of NLPR3, caspase-1, caspase-3, GSDMD and GSDME in RMS cells and skeletal muscle cells. **C** Western blot was used to detect the expression of NLPR3, caspase-1, caspase-3, GSDMD and GSDME in RMS cells and skeletal muscle cells.

To determine whether pyroptosis occurs in RMS, we first detected the expression of NLRP3, caspase-1, caspase-3, GSDMD and GSDME in RMS tissues and skeletal muscle tissues through IHC. NLRP3, caspase-1, GSDMD and GSDME were detected in the cytoplasm, and caspase-3 was detected in the cytoplasm and/or nucleus. The expression levels of NLRP3, caspase-1, caspase-3, GSDMD and GSDME were moderate to strongly positively expressed in most skeletal muscle tissues, while they were lowly expressed in RMS. The expression levels of them in RMS were significantly lower than those in the skeletal muscle tissues (Fig. 1A). We then examined the expression levels of NLRP3, caspase-1, caspase-3, GSDMD and GSDME in four different human cell lines and found that their mRNAs expression levels were significantly lower in the RD, RH30 and PLA-802 cells than in HSKMC cells (Fig. 1B). Their protein expression levels were also lower in the RMS cells than in the skeletal muscle cells (Fig. 1C). The above results indicated that the pyroptosis level is significantly downregulated in RMS cells.

### Nigericin-induced pyroptosis in RMS cell lines through the NLRP3/caspase-1/GSDMD pathway

**Fig. 2:**
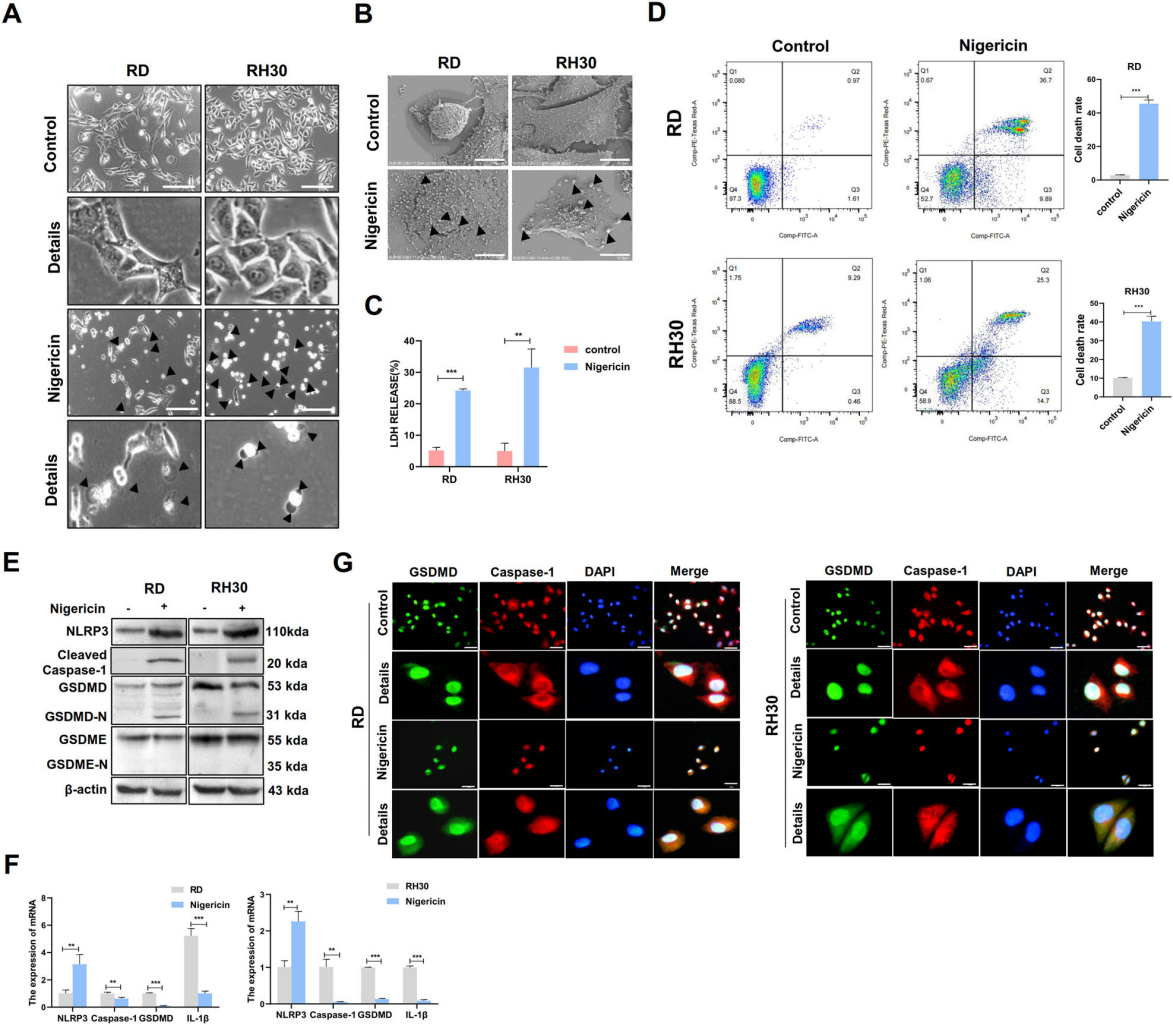
Nigericin-induced pyroptosis in RMS cells through the NLRP3/ caspase-1/GSDMD pathway. **A** Pyroptotic morphology after nigericin treatment under a light microscope. Arrows in the figure show pyroptotic vacuoles. Scale bar, 200 μm. **B** Pyroptotic morphology under an electron microscope. Arrows in the figure show pyroptotic vesicles. Scale bar, 10 μm. **C** ELISA was used to measure LDH production in the supernatant of RMS cells after nigericin treatment. **D** Flow cytometry revealed an increased proportion of pyroptosis after nigericin treatment. **E** Western blot was used to detect the expression of NLRP3, caspase-1, GSDMD and GSDME in RMS cells after nigericin treatment. **F** qRT-PCR was used to detect the expression of NLRP3, caspase-1, GSDMD, GSDME and IL-1β in RMS cells after nigericin treatment. **G** IF was used to detect the localisation of caspase-1 and GSDMD in RMS cells after nigericin treatment. Scale bar, 50 µm.

To elucidate nigericin whether induces RMS cell pyroptosis, we treated RD and RH30 cells with nigericin. Morphologic observation of the morphology of the RMS cells after nigericin treatment revealed the formation of pyroptotic vacuoles (Fig. 2A). Multiple microbubbles also appeared under electron microscopy (Fig. 2B). Pyroptotic cells can release cytoplasmic contents, such as LDH (Fig. 2C). Flow cytometry showed an increase in annexin-V and PI double-positive cells and the pyroptosis rate of RMS cells after nigericin treatment (Fig. 2D). These results suggested that nigericin can induce pyroptosis in RMS cells. To further confirm whether nigericin regulates RMS cell pyroptosis, we examined the alteration of pyroptosis-pathway molecules. The protein expression levels of NLRP3 and its downstream cleaved caspase-1 and GSDMD-N increased after nigericin treatment. However, GSDME-N was undetected (Fig. 2E). The mRNA expression of NLRP3 was also enhanced, and that of caspase-1, GSDMD and intracellular inflammatory factor IL-1 β decreased (Fig. 2F). IF assay on the cellular localisation of caspase-1 and GSDMD revealed that GSDMD was expressed in the nucleus and cytoplasm of RMS cells, and caspase-1 was primarily localised in the cytoplasm. However, caspase-1 underwent nuclear translocation from its original cytoplasmic expression into the nucleus after nigericin treatment. The GSDMD expression in the cytoplasm of RMS cells also increased, suggesting that nigericin induced the nuclear translocation of caspase-1 to cleave the GSDMD in the nucleus and release it into the cytoplasm (Fig. 2G). In summary, nigericin can induce pyroptosis through the NLRP3/caspase-1/GSDMD pathway.

### NSA can partially reverse the pyroptosis caused by nigericin

**Fig. 3:**
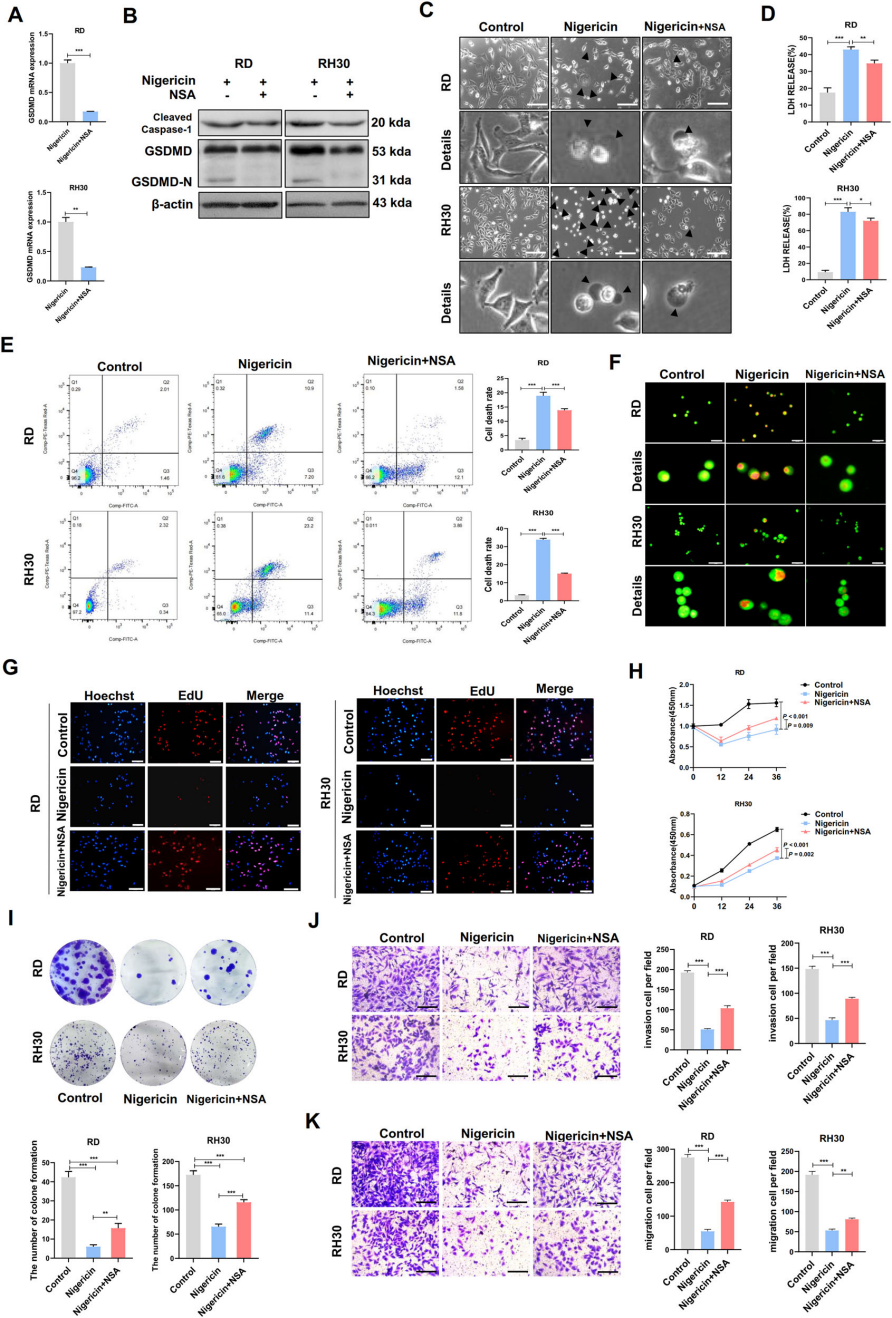
NSA reversed the NLRP3/caspase-1/GSDMD classical pyroptosis pathway caused by nigericin. **A** qRT-PCR was used to detect the mRNA expression of GSDMD in RMS cells treated with NSA and nigericin. **B** Western blot was used to detect the protein expression of pyroptosis-related genes in RMS cells by NSA treatment. **C** Pyroptotic vacuoles were observed under a light microscope after NSA treatment. Scale bar, 200 μm. **D** ELISA was used to measure LDH production in the RMS cell supernatant after NSA treatment. **E** Reduced percentage of pyroptosis after NSA treatment by flow cytometry. **F** AO staining assay was used to observe RMS cell death after NSA treatment. RD: Scale bar, 100 µm. RH30: Scale bar, 50 µm. **G** Enhanced proliferation of RMS cells after NSA treatment as detected by EdU assay. Scale bar, 50 µm. **H** CCK8 assay was used to detect RMS cell proliferation ability after NSA treatment. **I** Cloning assay was used to detect the proliferation of RMS cells after NSA treatment. **J**, **K** Transwell assay was used to detect the invasive and migratory ability of RMS cells after NSA treatment. Scale bar, 200 µm.

To explore the underlying mechanism, we used NSA, an inhibitor of GSDMD. NSA can directly bind to GSDMD without affecting GSDME, thereby inhibiting p30-GSDMD pore formation and pyroptotic cell death [22]. In addition, NSA can suppress the pyroptosis and necroptosis pathways to reduce intestinal inflammation for inflammatory bowel disease therapy [23]. We analysed its effect on the pyroptosis and biological behaviour of RMS cells. Compared with that treatment with nigericin alone, the mRNA expression of GSDMD in RMS cells was downregulated after with NSA+ nigericin (Fig. 3A). The protein expression of GSDMD-N also decreased, but that of NLRP3 and cleaved caspase-1 remained unchanged (Fig. 3B). In the NSA + nigericin group, the number of pyroptotic vacuoles decreased under light microscopy (Fig. 3C). We also observed a decrease in LDH production in the cell supernatant (Fig. 3D), pyroptosis degree and cell death (Fig. 3E). AO staining showed that cell death also decreased in the NSA + nigericin group (Fig. 3F). These results indicated that pyroptosis is partially suppressed after GSDMD inhibition.

We conducted a series of cell function experiments to further understand whether GSDMD inhibition affects the biological behaviour of RMS. The NSA +nigericin treatment group had more proliferating cells (Fig. 3G), stronger cell-proliferation ability (Fig. 3H) and more clonal clusters (Fig. 3I) than the nigericin group. The number of invading cells and migrating cells across the basement membrane in the NSA + nigericin treatment group also increased compared with those in the nigericin group (Fig. 3J, K). These findings indicated that inhibiting GSDMD can reverse the pyroptosis-related changes induced by nigericin and thus promote the proliferation, invasion and migration ability of RMS cells.

### Dactinomycin-induced pyroptosis in RMS cell lines through the caspase-3/GSDME pathway

**Fig. 4:**
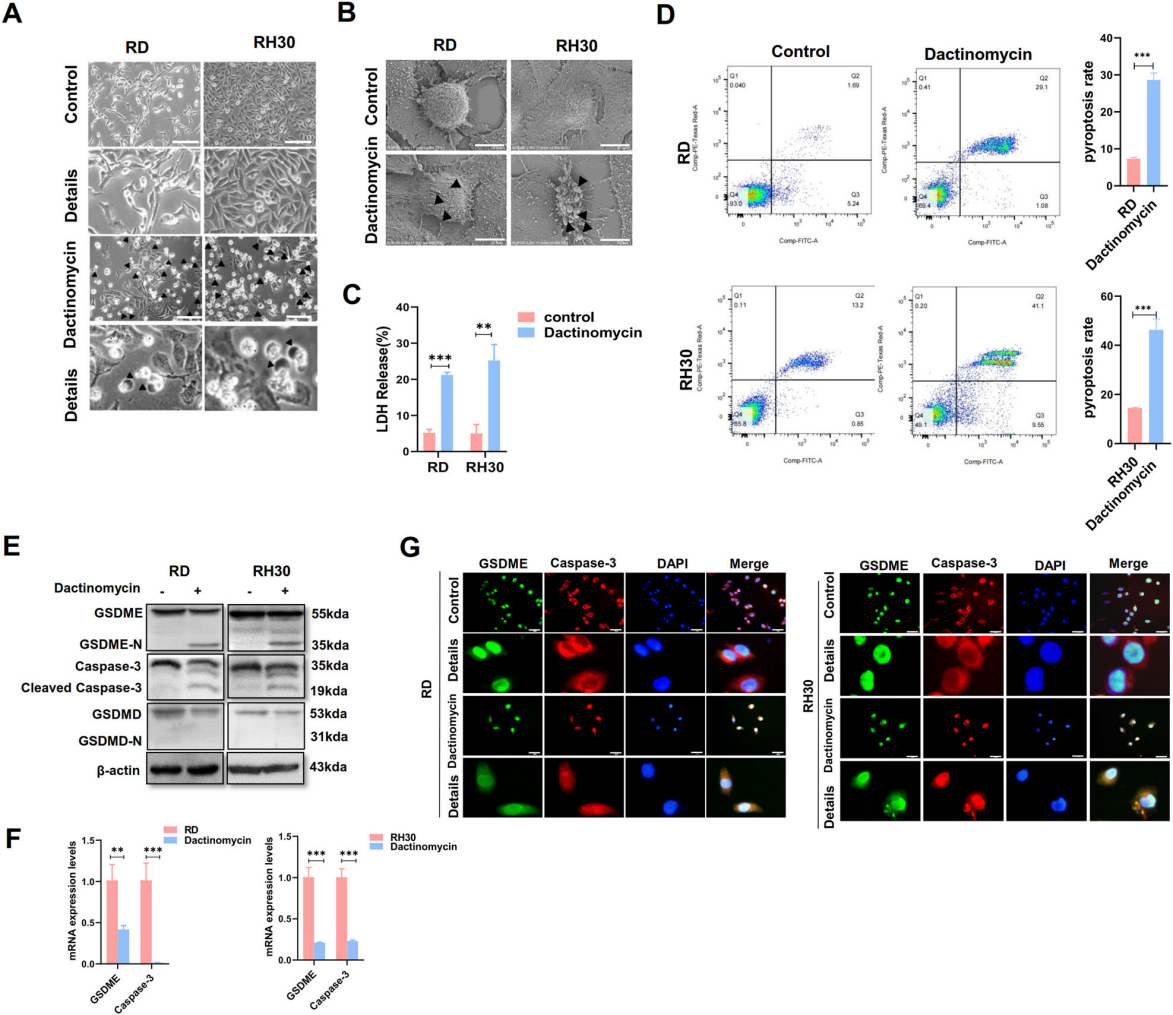
Dactinomycin-induced pyroptosis in RMS cells through the caspase-3/GSDME pathway. **A** Pyroptotic morphology after dactinomycin treatment under a light microscope. Arrows in the figure show pyroptotic vacuoles. Scale bar, 200 µm. **B** Pyroptotic morphology after dactinomycin treatment under an electron microscope. Arrows in the figure show pyroptotic vesicles. Scale bar, 10 µm. **C** ELISA results showed that dactinomycin increased the LDH content in the RMS cell supernatant. **D** Flow cytometry revealed an increased percentage of dactinomycin-induced pyroptosis. Western blot (**E**) and qRT-PCR (**F**) were used to detect the expression of caspase-3, GSDMD and GSDME in RMS cells after dactinomycin treatment. **G** IF was used to detect the localisation of caspase-3 and GSDME in RMS cells after dactinomycin treatment. Scale bar, 50 µm.

To determine whether dactinomycin affects RMS cell pyroptosis, we treated RD and RH30 cells with dactinomycin and confirmed the presence of pyroptotic vacuoles under a light microscope and multiple microbubbles under an electron microscope (Fig. 4A, B). Pyroptotic cells can release LDH (Fig. 4C). The RMS cells had a higher pyroptosis rate after dactinomycin treatment (Fig. 4D). The expression levels of cleaved caspase-3 and GSDME-N were upregulated after dactinomycin treatment, but GSDMD-N was undetected (Fig. 4E, F). IF assay results showed that dactinomycin induced caspase-3, which underwent nuclear translocation to cleave the GSDME in the nucleus and release it into the cytoplasm (Fig. 4G). These results suggested that dactinomycin can induce pyroptosis via the caspase-3/GSDME pathway.

### SiGSDME can partially reverse pyroptosis caused by dactinomycin

**Fig. 5:**
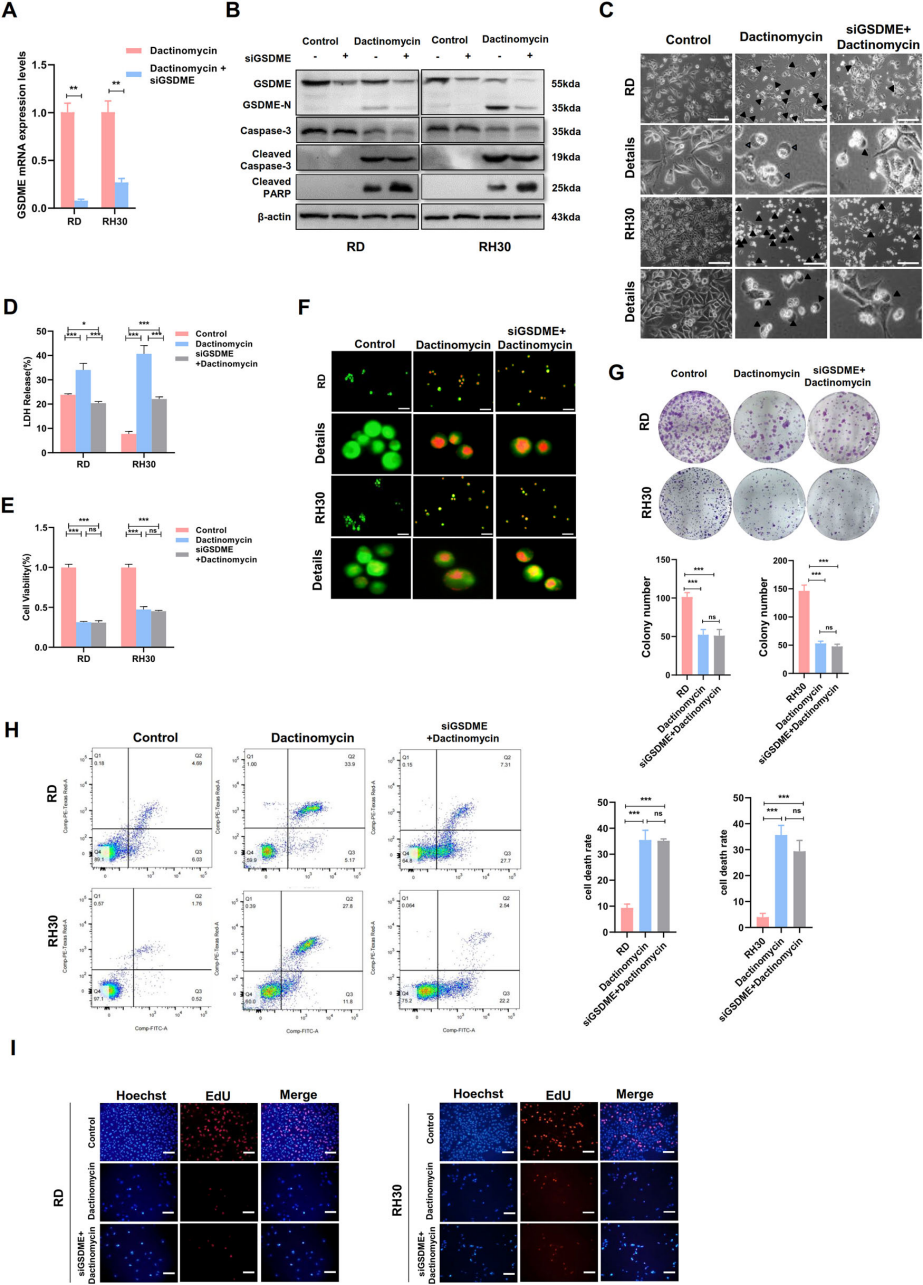
siGSDME reversed the changes in caspase-3/GSDME pyroptosis-related genes caused by dactinomycin and prompted the conversion of pyroptosis into apoptosis. **A** qRT-PCR was used to detect the mRNA expression of GSDME in RMS cells treated with siGSDME and dactinomycin. **B** Western blot showed a decrease in GSDME-N terminal and an increase in cleaved-PARP expression induced by dactinomycin after siGSDME. **C** Pyroptotic morphology in the siGSDME treatment group and dactinomycin + siGSDME treatment group under a light microscope. Scale bar, 200 μm. **D** Detection of LDH release from cell supernatants by ELISA. **E** Cell viability detected by MTT assay. **F** AO staining assay demonstrated no significant effect on dactinomycin-induced cell death after siGSDME treatment. Scale bar, 100 µm. **G** Cloning assay was used to detect the proliferation of RMS cells after siGSDME treatment. **H** Flow cytometry was used to detect RMS cell apoptosis in the siGSDME treatment and dactinomycin + siGSDME treatment groups. **I** EdU assay was used to detect the proliferation of RMS cells in the siGSDME treatment group and dactinomycin + siGSDME treatment group. Scale bar, 50 µm.

To understand the impact of GSDME on pyroptosis, we first successfully knocked down GSDME (Fig. 5A). In the RMS cells treated with siGSDME, dactinomycin still caused caspase-3 activation, but GSDME cleavage was significantly reduced, and GSDME-N release decreased. Conversely, the expression of apoptosis-associated molecule cleaved-PARP increased (Fig. 5B). PARP is a DNA repair enzyme belonging to the substrate of caspase, the core molecule of cell apoptosis. When cells undergo apoptosis, PARP is cleaved to form cleaved PARP. Our results indicated that knocking down GSDME in RMS cells may affect dactinomycin-induced cell pyroptosis and apoptosis.

We subsequently confirmed the effect of GSDME on pyroptosis or apoptosis and found pyroptotic vacuoles (Fig. 5C) and LDH-released products decreased in the dactinomycin + siGSDME treatment group compared with those in the dactinomycin treatment group (Fig. 5D). However, MTT and AO assays showed no significant change in dactinomycin-induced RMS cell death after GSDME knockdown (Fig. 5E, F). The proliferation of RMS cells was not reduced after siGSDME treatment (Fig. 5G). The dactinomycin-induced pyroptosis also decreased and apoptosis increased after GSDME knockdown, so the cell death rate did not significantly change (Fig. 5H). EdU experiment showed that knocking down GSDME had no effect on cell proliferation (Fig. 5I). siGSDME did not affect the ability of dactinomycin to inhibit RMS cell invasion and migration (Fig. S1). In summary, GSDME knockdown converts dactinomycin-induced pyroptosis into apoptosis but does not affect the malignant biological behaviour of RMS.

### GEFT-Rac1/Cdc42 was involved in the regulation of the GSDMD and GSDME pyroptosis signalling pathway

**Fig. 6:**
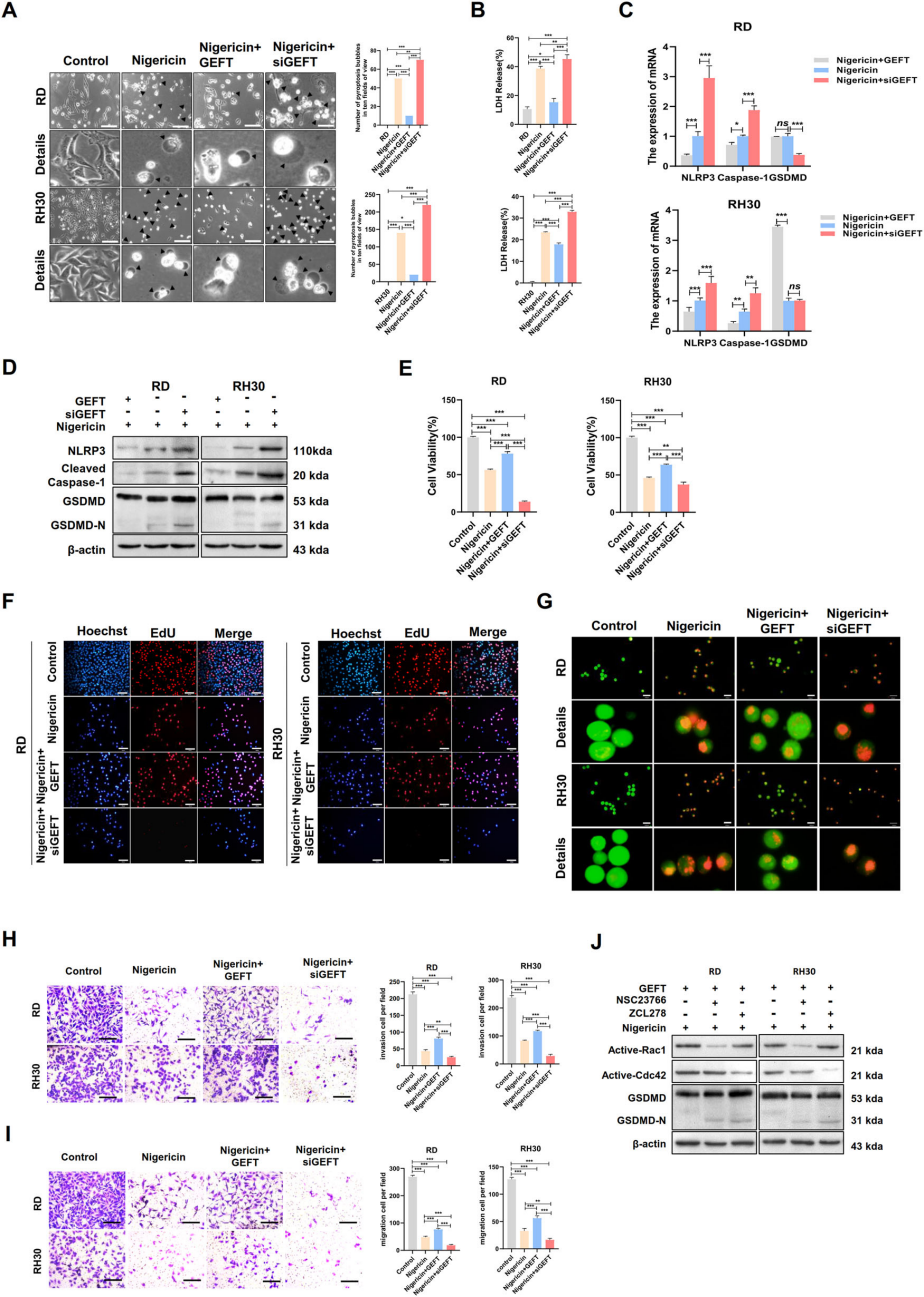
GEFT-Rac1/Cdc42 was involved in the regulation of RMS cell pyroptosis by the GSDMD signalling pathway. **A** Pyroptotic morphology in the control, nigericin treatment, GEFT + nigericin treatment, and siGEFT + nigericin treatment groups under a light microscope. Scale bar, 200 μm. **B** LDH release levels in each group of (**A)** were detected by ELISA. **C** Expression of NLRP3, caspase-1 and GSDMD mRNA in the nigericin, GEFT + nigericin and siGEFT + nigericin treatment groups as detected by qRT-PCR. **D** Western blot detecting the expression of NLRP3, caspase-1 and GSDMD in each group of (**C**). **E** MTT assay was used to detect RMS cell viability in each group of (**A**). **F** EdU assay was used to detect the proliferative capacity of RMS cells in each group of (**A**). Scale bar, 50 µm. **G** AO assay was used to detect RMS cell death in each group of (**A**). Scale bar, 100 µm. **H**, **I** Transwell assay was used to detect the invasive and migratory ability of RMS cells in each group of (**A**). Scale bar, 200 µm. **J** Western blot was used to detect the protein expression of pyroptosis-related genes in RMS cells after the inhibition of Rac1 or Cdc42.

Our group screened GEFT and found its close relation to the development of RMS [24]. RD and RH30 cells were treated with nigericin or dactinomycin and siGEFT and stably overexpressed with GEFT, respectively, to further clarify the relationship of GEFT and GSDMs signalling pathway. Compared with those in the nigericin treatment group, the pyroptotic vacuoles (Fig. 6A) and LDH production group (Fig. 6B) decreased in the GEFT + nigericin treatment group and increased in the siGEFT + nigericin treatment group. The mRNA and protein expression levels of NLRP3, cleaved-caspase-1 and GSDMD significantly decreased in the overexpressed GEFT+nigericin group (Fig. 6C, D). Moreover, nigericin can inhibit the cell viability and proliferation of RMS cells, and GEFT can partially restore this phenomenon, the combination of siGEFT and nigericin significantly inhibited cell viability and proliferation (Fig. 6E, F) and promoted RMS cell death (Fig. 6G), exerting a strongly inhibitory effect (Fig. 6H, I). These results indicated that GEFT can inhibit nigericin-induced pyroptosis and changes in cellular biological behaviour. Our preliminary research has demonstrated that GEFT can directly bind to Rac1 and Cdc42 to inhibit autophagy and apoptosis in RMS [25]. We further investigated whether GEFT also affects the pyroptosis pathway through Rac1/Cdc42. We found that GSDMD expression increased after the inhibition of Rac1/Cdc42 with NSC23766, an inhibitor of Rac1, or ZCL278, an inhibitor of Cdc42 (Fig. 6J). Thus, Rac1/Cdc42 can negatively regulate the GSDMD-mediated pyroptosis signalling pathway. The above results suggested that GEFT-Rac1/Cdc42 can inhibit RMS pyroptosis and its biological behaviour by regulating the GSDMD signalling pathway. GEFT can reverse drug-induced pyroptosis, and interference with GEFT increases drug sensitivity.

**Fig. 7:**
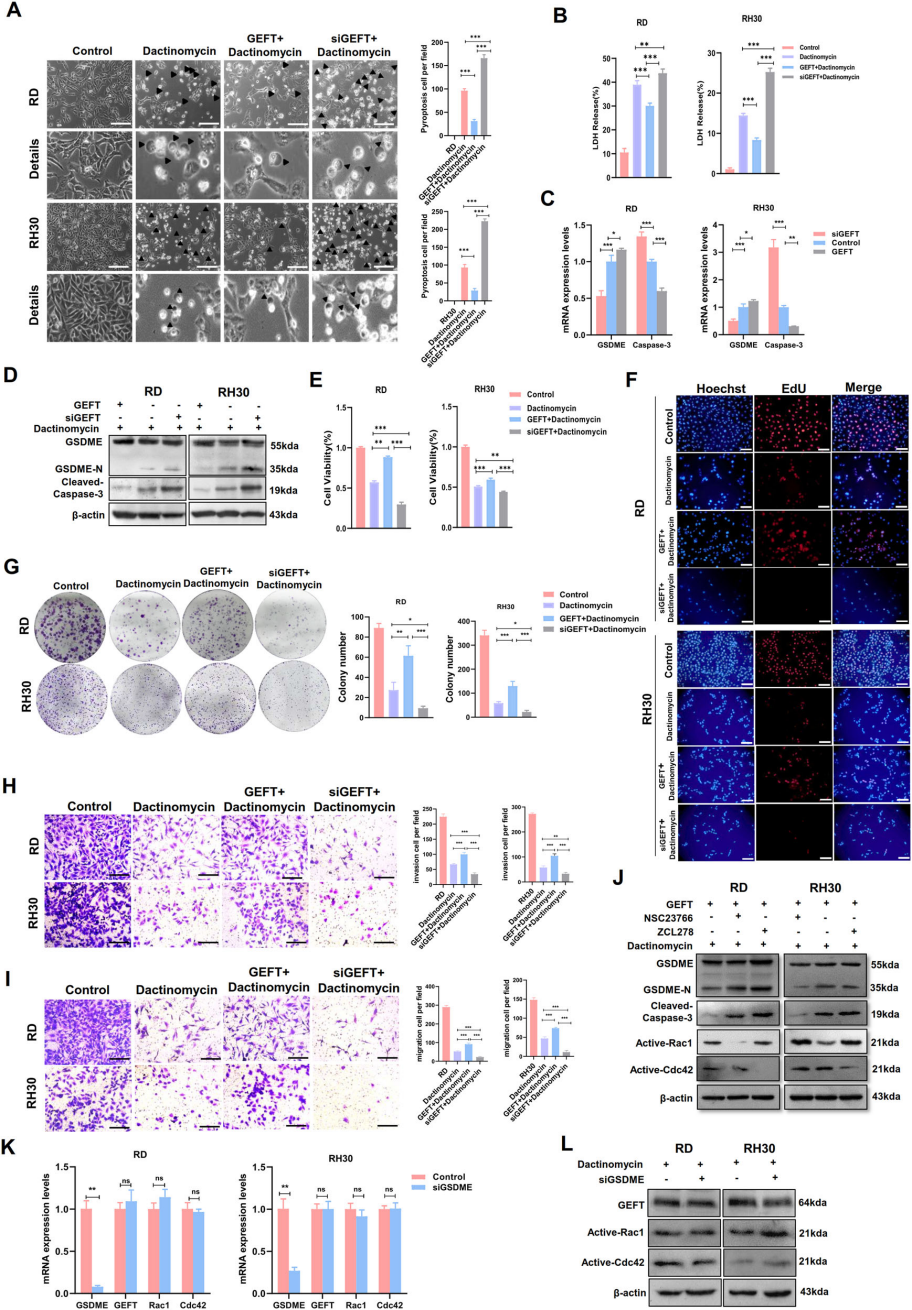
GEFT-Rac1/Cdc42 was involved in the regulation of RMS cell pyroptosis by the GSDME signalling pathway. **A** Pyroptotic morphology in the control group, dactinomycin treatment group, GEFT+ dactinomycin treatment group and siGEFT + dactinomycin treatment group under the light microscope. Scale bar, 200 μm. **B** LDH release levels in each group of (**A)** detected by ELISA. **C** mRNA expression of caspase-3 and GSDME in dactinomycin treatment, GEFT + dactinomycin treatment and siGEFT+dactinomycin treatment group by qRT-PCR. **D** Western blot was used to detect the expression of caspase-3 and GSDME in each group of (**C**). **E** MTT assay was used to detect RMS cell viability in each group of (**A**). **F** EdU assay was used to detect the proliferative capacity of RMS cells in each group of (**A**). Scale bar, 50 µm. **G** Cloning assay was used to detect the proliferation of RMS cells. **H**, **I** Transwell assay was used to detect the invasive and migratory ability of RMS cells. Scale bar, 200 µm. **J** Western blot was used to detect the protein expression of pyroptosis-related genes in RMS cells after NSC23766 or ZCL278 treatment. qRT-PCR (**K**) and western blot (**L**) were used to detect the expression of GSDME, GEFT, Rac1 and Cdc42.

Compared with those in the dactinomycin treatment group, the pyroptotic vacuoles (Fig. 7A) and LDH production (Fig. 7B) decreased in the overexpressed GEFT+ dactinomycin treatment group and increased in the siGEFT + dactinomycin treatment group. Caspase-3 mRNA and protein expression and GSDME expression significantly decreased in the overexpressed GEFT+ dactinomycin group (Fig. 7C, D). Dactinomycin can inhibit the cell viability and proliferation of RMS cells, and GEFT can partially restore this phenomenon, the combined application of siGEFT and dactinomycin exerted the most significant inhibition of cell viability and proliferation (Fig. 7E–G) and had a stronger effect than treatment with dactinomycin alone (Fig. 7H, I). GEFT inhibited the GSDME pyroptosis pathway by activating the Rac1/Cdc42 pathway (Fig. 7J). Inhibiting GSDME did not affect the expression of GEFT, Rac1 and Cdc42 (Fig. 7K, L). The above results suggested that GEFT-Rac1/Cdc42 inhibits RMS cell pyroptosis by regulating the GSDME signalling pathway, and interfering with GEFT increases the sensitivity of chemotherapeutic agents.

Our results suggested that GEFT-Rac1/Cdc42 is involved the regulation of the NLRP3/caspase-1/GSDMD canonical inflammasome and caspase-3/GSDME pyroptosis signalling pathways. The combination of interference GEFT and chemotherapy drugs can improve drug sensitivity.

### Animal study

**Fig. 8:**
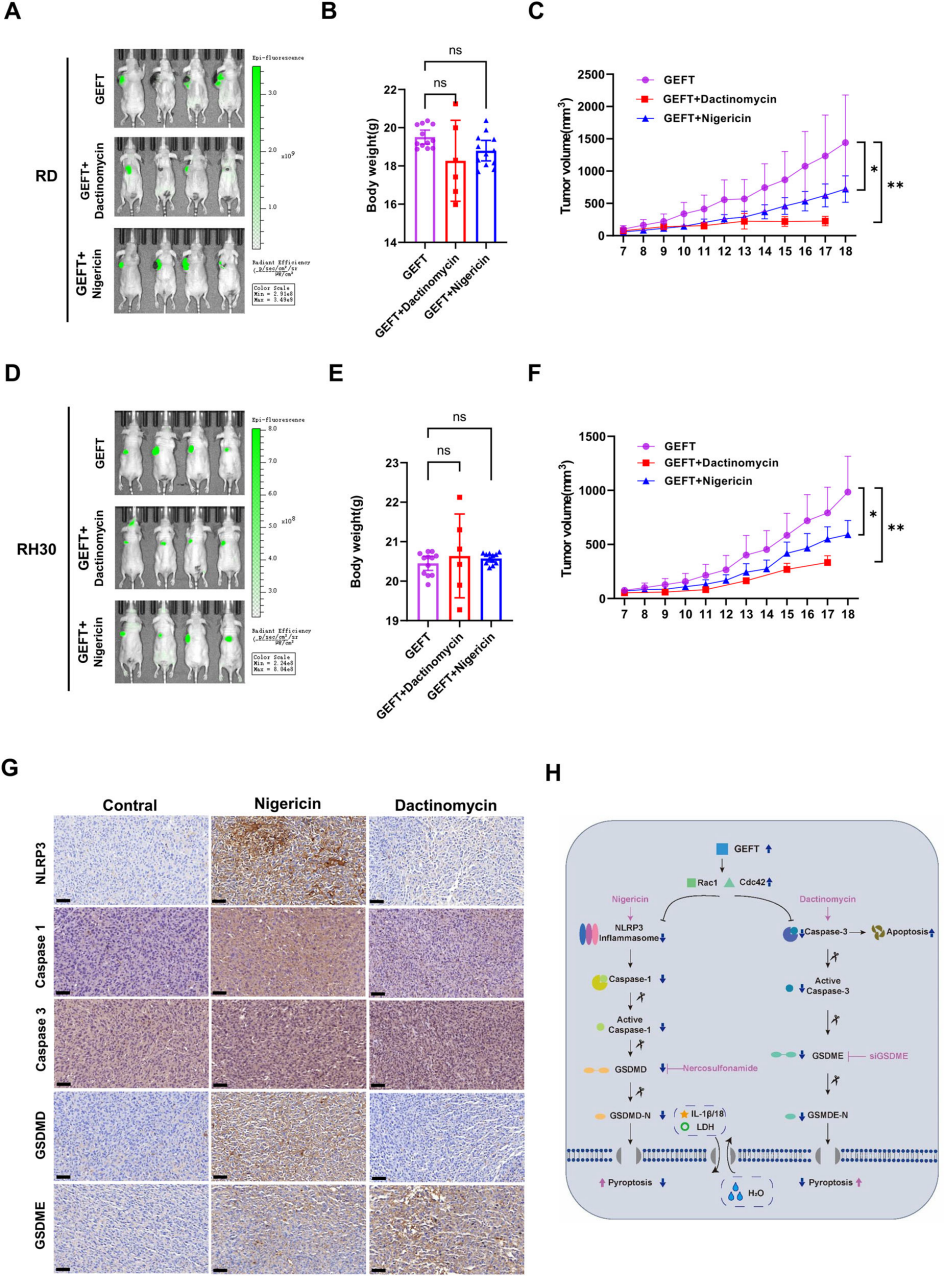
Nigericin induced the NLRP3/caspase-1/GSDMD pathway in vivo, and dactinomycin induced the caspase-3/GSDME pathway in vivo to promote pyroptosis. RD cell lines with stable GEFT were treated with different drugs, and whole-body GFP imaging after executing the mouse (**A**), body weight curve of nude mice (**B**) and growth curves of the xenografted tumours (**C**). RH30 cell lines with stable GEFT were treated with different drugs, and whole-body GFP imaging after executing the mouse (**D**), body weight curve of nude mice (**E**) and growth curves of the xenografted tumours (**F**). **G** IHC was used to detect the expression of NLRP3, caspase-1, caspase-3, GSDMD and GSDME in mouse tumour tissue. Scale bar, 100 µm. **H** Diagram of the molecular mechanism of GEFT regulating GSDMD/GSDME pyroptosis signalling pathway to promote drug resistance in RMS.

RD and RH-30 cells stably transfected with lentivirus GEFT were injected into nude mice to further clarify the therapeutic effect of drugs. After tumour cells were injected for 7 days, the tumour volume grew to 50 mm^3^ and the intraperitoneal injection of nigericin or dactinomycin was performed. Compared with that in the RD + GEFT group, the tumour volume significantly decreased in the nigericin injection group from the 4th day and in the dactinomycin injection group from the 3rd day (*p* < 0.05). Body weight was inhibited to a certain extent, but no statistical significance was found relative to the control group (Fig. 8A–C). Compared with that in the RH30 + GEFT group, the tumour volume significantly decreased in the nigericin injection group from the 8th day and in the dactinomycin injection group from the 2nd day (*p* < 0.05). The differences in body weight had no statistical significance relative to the control group (Fig. 8D–F). After 11 days of drug injection, all the mice were euthanized, and tumours were removed for IHC analysis. The expression levels of NLRP3, caspase-1 and GSDMD in the nigericin injection group were significantly higher than those in the untreated group. The expression levels of caspase-3 and GSDME in the dactinomycin injection group were significantly higher than those in the untreated group (Fig. 8G). The molecular mechanism diagram of GEFT regulating the GSDMD/GSDME pyroptosis signalling pathway to promote drug resistance in RMS is shown in Fig. 8H.

## Discussion

Pyroptosis is a programmed cell death mediated by the GSDM family of proteins, which includes GSDMA‑GSDME and PJVK. The GSDM family of proteins have similar structures. GSDM is cleaved into C- and N-terminal domains, with the latter perforating the plasma membrane to execute pyroptosis [26]. Pyroptosis has four main signalling pathways, namely, canonical inflammasome, non-canonical inflammasome, apoptotic caspases-mediated, and granzymes-based pathways. GSDM proteins cleaved by upstream caspases or granzymes serve as the final executioners in these signalling pathways. If GSDM is not expressed, caspase protease activation alone cannot induce cell pyroptosis [27]. As an important immune response, pyroptosis is involved in the occurrence and development of infectious diseases and metabolic diseases. In recent years, the mechanism of tumour cell pyroptosis has become a research hotspot. Promoting tumour cell pyroptosis is expected to become the direction of tumour treatment.

GSDMD is located on human chromosome 8q24.3. GSDMD‑induced pyroptosis is cleaved and activated by caspase‑1, ‑4, ‑5, ‑8 and ‑11. Caspase‑1 activates GSDMD through inflammasome complexes, such as NLRP1, NLRP3 and AIM2 [28]. Almost all human organs and tissues can express the protein of GSDMD, and GSDMD participates in various biological processes [29]. GSDMD is downregulated or silenced in some tumours but upregulated in other tumours. In gastric cancer, GSDMD expression decreases, markedly promoting the proliferation of tumours and accelerating S/G2 transition by activating extracellular signal-regulated kinase and regulating cell cycle-related proteins [30]. In renal cell carcinoma, BRD4 expression levels are markedly upregulated, and pyroptosis-associated proteins are significantly reduced. BRD4 inhibition prevents cell proliferation and epithelial–mesenchymal transition, exerting an antitumour effect by activating the NF-Κb/NLRP3/caspase-1 pyroptosis signalling pathway [31]. In hepatocellular carcinoma, sorcin is considerably upregulated and caspase-1, GSDMD-N and IL-1β are significantly decreased. Sorcin knockdown can activate pyroptosis [32]. In oral squamous cell carcinoma cells, anthocyanin could reduce the viability, inhibit the migration and invasion abilities and enhance the expression of NLRP3, caspase-1 and IL-1β. Anthocyanin-activated pyroptosis was suppressed upon the administration of caspase-1 inhibitors [33]. However, GSDMD is upregulated in glioma compared with that in non-tumour brain tissues. High GSDMD expression is significantly associated with WHO grade IV and short overall survival. GSDMD expression also increases in a time-dependent manner after treatment with temozolomide [34]. Nigericin causes triple-negative breast cancer cell death by inducing concurrent caspase-1/GSDMD-mediated pyroptosis and promoting the infiltration and activation of T cells, making it a promising antitumour agent, especially when combined with anti-PD-1 antibody treatment [35]. Decitabine upregulates GSDMD, which is then cleaved by NLRP3 and caspase-1, both of which are activated by nigericin. Thus, effective cancer cell pyroptosis is achieved, and a significant systemic antitumour immunity is induced [36]. In our study, we found for the first time that the pyroptosis levels are significantly low in RMS tissues and cells. Furthermore, we detected for the first time how nigericin induces RMS cells pyroptosis: by activating the NLRP3/caspase-1/GSDMD signalling pathway.

GSDME is located on human chromosome 7q15. GSDME‑induced pyroptosis primarily relies on the activation of caspase‑3. Activated caspase‑3 cleaves GSDME and induces pyroptosis instead of apoptosis. Granzyme B may also directly cleave and activate GSDME [37]. USP48 can affect the expression of GSDME through physical interaction with GSDME and does not affect the activation and cleavage of GSDME by caspase-3 [38]. Dioscin can induce G2/M‐phase arrest and apoptosis through the JNK/p38 pathway and activate GSDME‐dependent pyroptosis in osteosarcoma. Accordingly, osteosarcoma growth is inhibited [39]. In pancreatic ductal adenocarcinoma, chemotherapeutic drugs induce concurrent pyroptosis and apoptosis, and GSDME is cleaved by activated caspase-3. GSDME knockdown converts pyroptosis into apoptosis, decreases invasion and migration and enhances the sensitivity of carcinoma cells to chemotherapy [40]. In oral squamous cell carcinoma, decitabine combined with cisplatin and immune-checkpoint inhibitors can amplify the immunotherapies outcomes, and the activation of GSDME can improve the sensitivity of chemotherapeutics and activate inflammatory tumour-cell pyroptosis [41]. In ovarian cancer, CBL0137 can activate ROS/BAX signalling and promote caspase-3/GSDME-dependent pyroptosis [42]. Although dactinomycin, in combination with other chemotherapy drugs, is included in RMS treatment, the relationship between dactinomycin and pyroptosis in RMS has not been reported. Herein, we studied for the first time the mechanism of dactinomycin for inducing pyroptosis in RMS. Our results showed that dactinomycin induces pyroptosis by activating the caspase-3/GSDME signalling pathway. GSDME knockdown can convert dactinomycin-induced pyroptosis into apoptosis but does not affect the malignant biological behaviour of RMS.

Our preliminary research has shown that increased GEFT expression may be involved in the pathogenesis of RMS by activating the Rac1/Cdc42 signalling pathway and miR-144-3p/mTOR signalling pathway [5, 43]. However, whether the GEFT pathway can regulate pyroptosis in RMS cells has not yet been studied. We further investigated the relationship between the GEFT pathway and pyroptosis and found that GEFT-Rac1/Cdc42 can inhibit RMS pyroptosis by regulating the GSDMD and GSDME signalling pathway, that GEFT can also reverse the drug-induced pyroptosis, and interference with this molecule can increase the sensitivity of nigericin and dactinomycin. Our study revealed the regulatory role of GEFT in pyroptosis and its molecular mechanism.

In conclusion, our study investigated for the first time the pyroptosis level in RMS. The pyroptosis-associated molecules were initially silenced or downregulated in RMS. We further confirmed that nigericin and dactinomycin promoted cell pyroptosis in RMS. Nigericin and dactinomycin induced pyroptosis through the caspase-1/GSDMD classical pyroptosis pathway and caspase-3/GSDME apoptotic caspase-mediated pyroptosis pathway, respectively. Moreover, interference with GEFT combined with nigericin or dactinomycin can effectively improve drug sensitivity. Our research still has limitations. We only studied how the GEFT pathway can affect pyroptosis through the NLPR3/caspase-1/GSDMD and caspase-3/GSDME pathways. The specific molecular mechanism still requires further investigation.

## Supplementary information

supplementary materials

## Data Availability

All data generated during this study are included in this published article and its Supplementary Information files.
