# Supplementary material for: GEFT inhibits the GSDM-mediated proptosis signalling pathway, promoting the progression and drug resistance of rhabdomyosarcoma
Source: Cell Death Dis. 2024 Nov 30;15(11):867. doi: 10.1038/s41419-024-07243-y (PMC11608370; doi:10.1038/s41419-024-07243-y)

Table S1 Primer and interference fragment sequence

Primers	sequence
NLRP3-F	CGTGAGTCCCATTAAGATGGAGT
NLRP3-R	CCCGACAGTGGATATAGAACAGA
Caspase-1-F	TTTCCGCAAGGTTCGATTTTCA
Caspase-1-R	GGCATCTGCGCTCTACCATC
Caspase-3-F	ATGGAGAACAATAAAACCT
Caspase-3-R	CTAGTGATAAAAGTAGAGTTC
GSDMD-F	TGAATGTGTACTCGCTGAGTGTGG
GSDMD-R	CAGCTGCTGCAGGACTTTGTG
GSDME-F	CCCAGGATGGACCATTAAGTGT
GSDME-R	GGTTCCAGGACCATGAGTAGTT
IL-1β-F	AGCTACGAATCTCCGACCAC
IL-1β-R	CGTTATCCCATGTGTCGAAGAA
GEFT-F	CCCAAGTCAGAGCATGTGGT
GEFT-R	CCCTCAAATCCCCGCAATCT
Rac1-F	CTCAAGACAGTGTTTGACGAAG
Rac1-R	TTACAACAGCAGGCATTTTCTC
Cdc42-F	GCTTGTTGGGACTCAAATTGAT
Cdc42-R	CCTTTCTGTGTAAGTGCAGAAC
GAPDH-F	TGCACCACCAACTGCTTAGC
GAPDH-R	GGCATGGACTGTGGTCATGAG
si-GEFT-F	CCUCUUGCCACUGGAUAAATT
si-GEFT-R	UUUAUCCAGUGGCAAGAGGTT
si-GSDME-F	GGUCCUAUUUGAUGAUGAATT
si-GSDME-R	UUCAUCAUCAAAUAGGACCGC

Supplementary

Fig. S1 Knocking down GSDME has no effect on the invasion and migration ability of RMS cells. Transwell assay was used to detect RMS cell invasion **(A)** and migration **(B)** after siGSDME treatment. Scale bar, 200 μm.

A

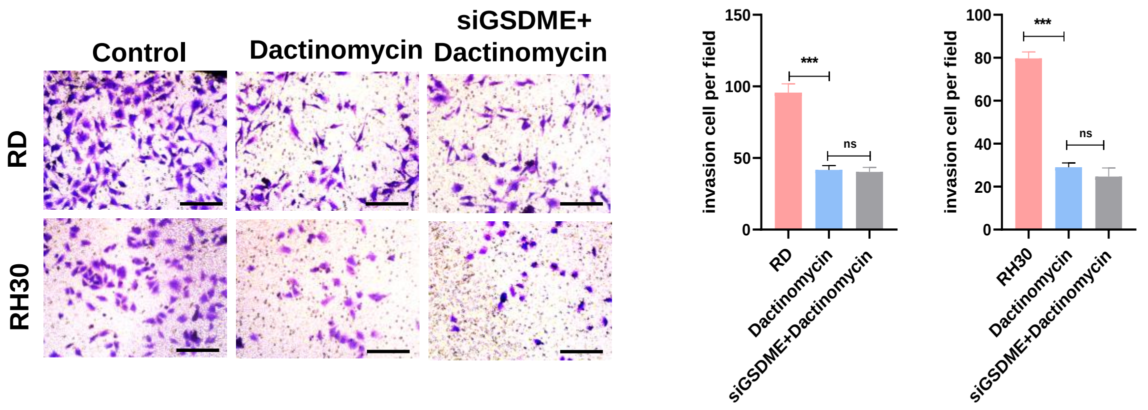
Control
Dactinomycin
siGSDME+
Dactinomycin
RD
RH30
invasion cell per field
150
100
50
0
***
ns
RD
Dactinomycin
siGSDME+Dactinomycin
invasion cell per field
100
80
60
40
20
0
***
ns
RH30
Dactinomycin
siGSDME+Dactinomycin

B

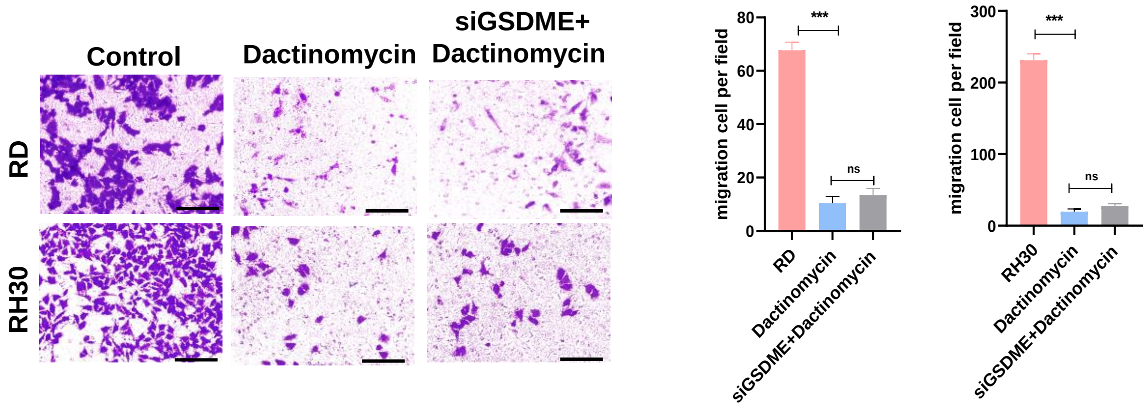
siGSDME+
Control
Dactinomycin
Dactinomycin
RD
RH30
migration cell per field
80
60
40
20
0
***
ns
RD
Dactinomycin
siGSDME+Dactinomycin
migration cell per field
300
200
100
0
***
ns
RH30
Dactinomycin
siGSDME+Dactinomycin

Fig.1C

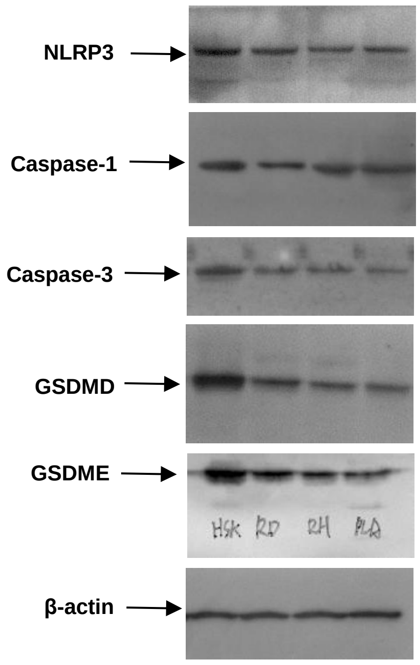

Fig.2E(RD)

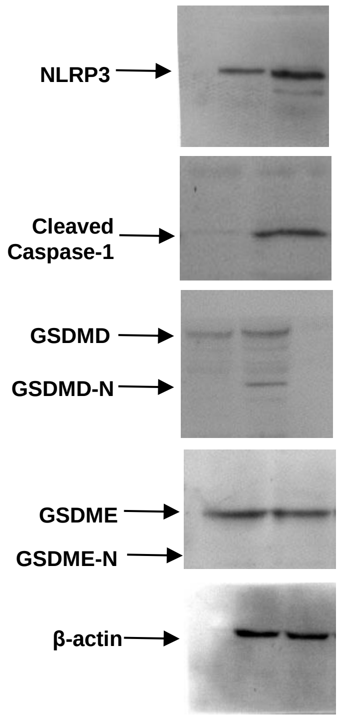

Fig.2E(RH30)

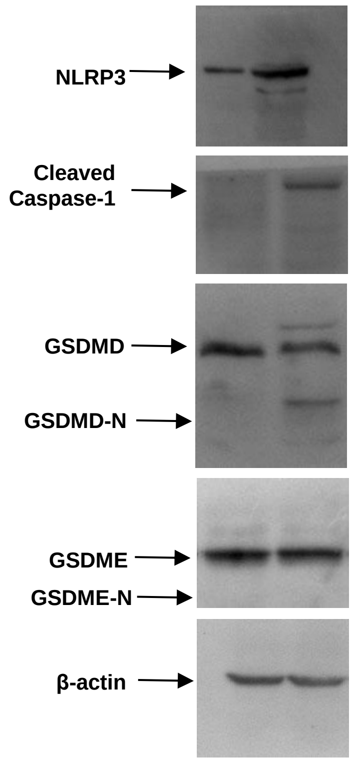

Fig.3B(RD)

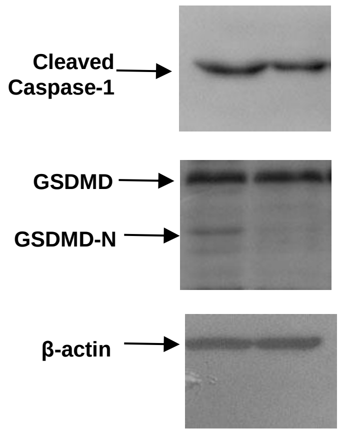

Fig.3B(RH30)

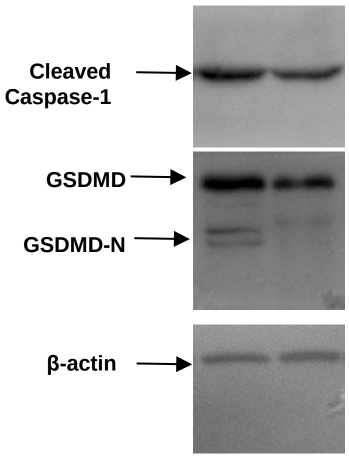

Fig.4E(RD)

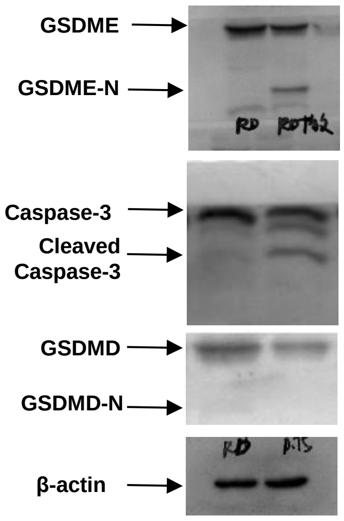

Fig.4E(RH30)

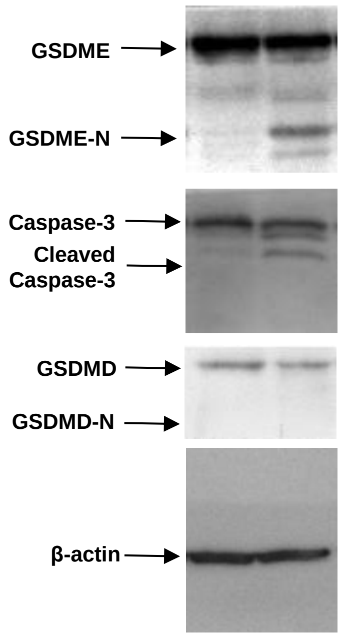

Fig.5B(RD)

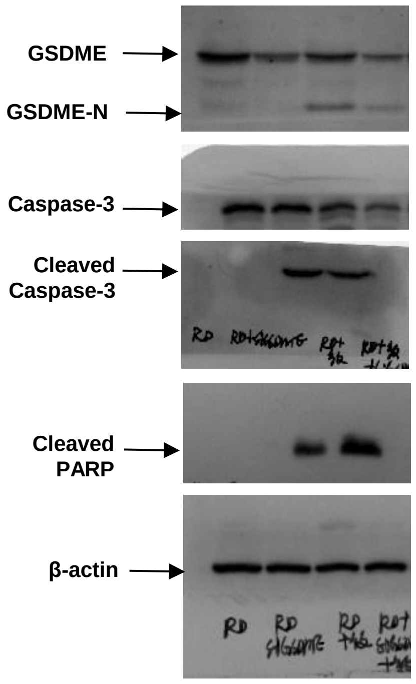

Fig.5B(RH30)

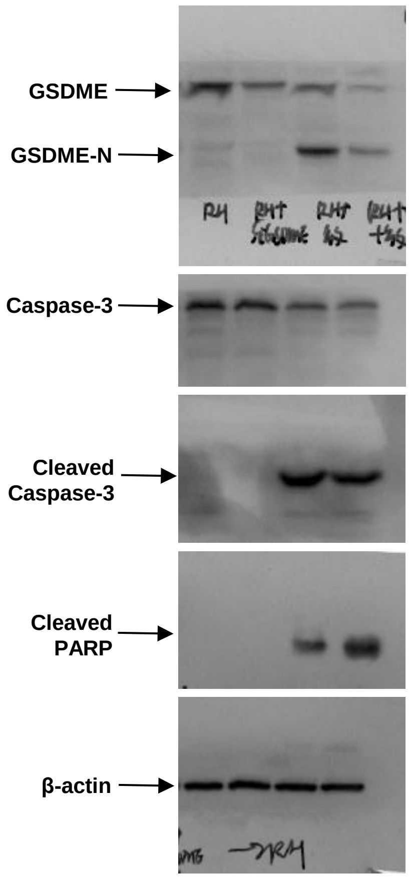

Fig.6D(RD)

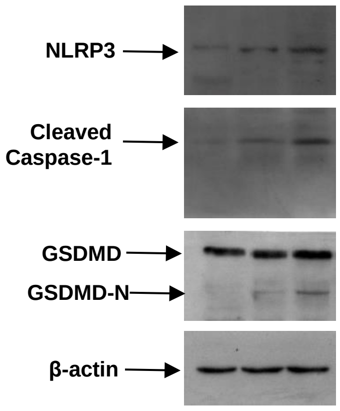

Fig.6D(RH30)

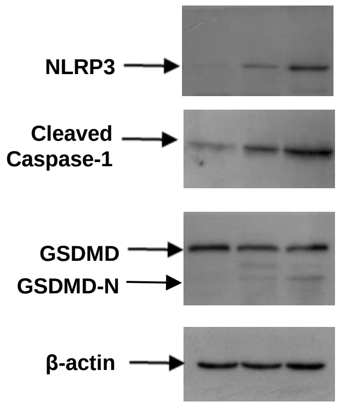

Fig.6J(RD)

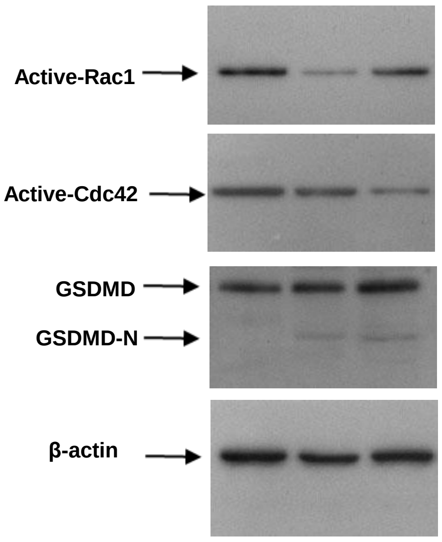

Fig.6J(RH30)

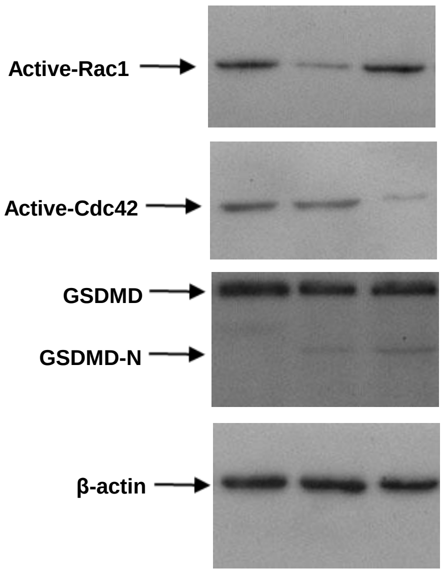

Fig.7D(RD)

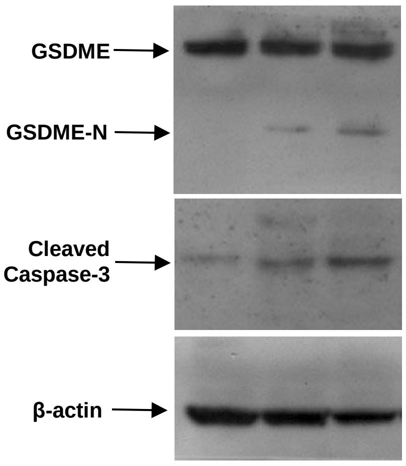

Fig.7D(RH30)

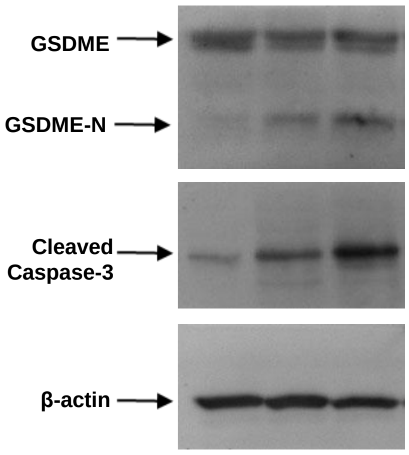

Fig.7J(RD)

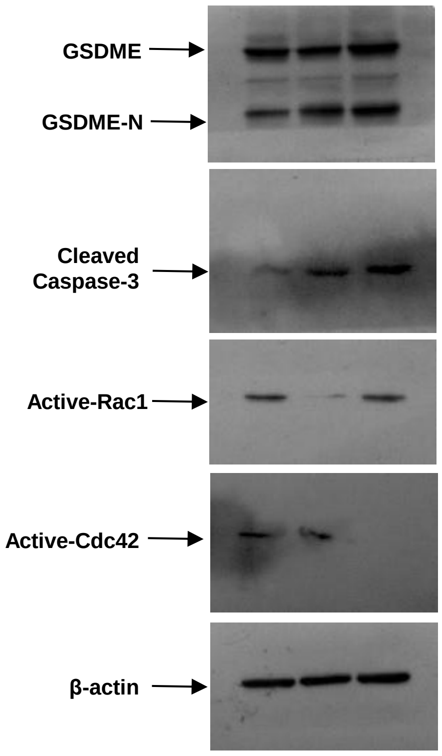

Fig.7J(RH30)

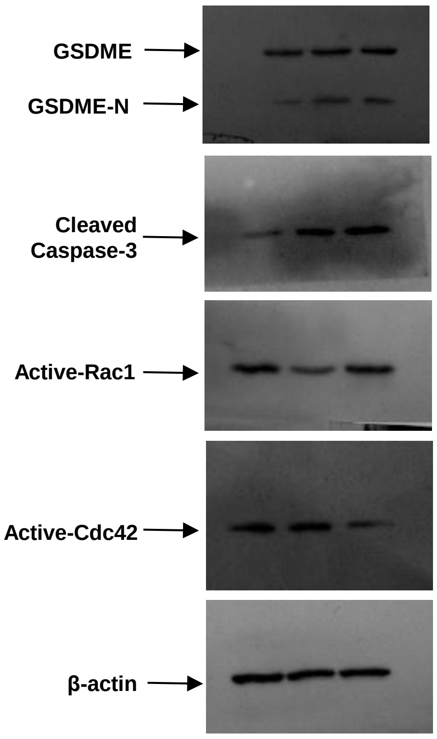

Fig.7L(RD)

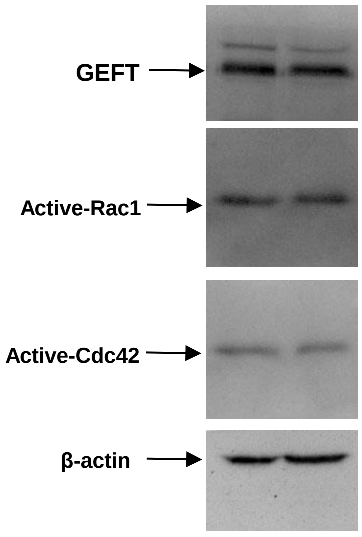

Fig.7L(RH30)

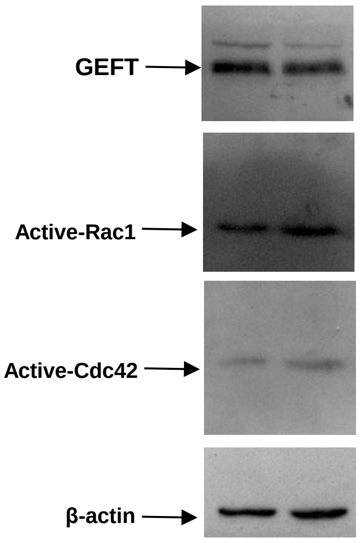

Supplement: Supplementary file 1 — supplementary materials [file 41419_2024_7243_MOESM1_ESM.pdf]
